# Changes in trust and the use of Korean medicine in South Korea: a comparison of surveys in 2011 and 2014

**DOI:** 10.1186/s12906-017-1969-8

**Published:** 2017-09-16

**Authors:** Soohyun Kwon, Shinhee Heo, Dongjun Kim, Seunghyun Kang, Jong-Min Woo

**Affiliations:** 1 Guideline Center for Korean Medicine, National Institute of Korean Medicine, Namsan Square 173, Toegye-ro, Jung-gu, Seoul, 04554 South Korea; 2 School of Korean Medicine, Pusan National University, Yangsan, 50612 South Korea; 3 R&D Strategy and Planning Team, Korea Institute of Radiological and Medical Sciences, 75 Nowon-Ro, Nowon-Gu, Seoul, 01812 South Korea; 4 Policy Division, Korea Institute of Oriental Medicine, 1672 Yuseongdae-ro, Yuseong-gu, Daejeon 34054 South Korea

**Keywords:** Korean medicine, Periodic survey, Prevalence, Perception, South Korea

## Abstract

**Background:**

Korean medicine (KM) has been widely used in Korea. This study aimed to assess the general perceptions of KM, to investigate the patterns of its usage in 2014, and to compare the results with those of an earlier survey from 2011.

**Methods:**

A cross-sectional study was conducted with 1000 Korean people. The questionnaire included items regarding trust in KM, reasons for distrust of KM, and visit frequency to KM clinics. This study used methods consistent with those of a 2011 survey to examine changes in attitudes over 3 years.

**Results:**

Despite high rates of trust in KM, the visit frequency decreased from 69.3% in 2011 to 63.2% in 2014. Usage among young adults (in their 20s and 30s) was significantly reduced compared to all other age groups. The KM modality most commonly used by participants was acupuncture, whereas the use of moxibustion and cupping therapies has decreased since 2011. Men and women were most likely to distrust KM due to a “lack of scientific evidence” (59.3%) and “suspicion of KM safety” (47.4%), respectively.

**Conclusions:**

The findings suggested that KM use and trust in KM were slightly lower in 2014 than in 2011. The decreases were most notable among individuals in their 30s and in the use of moxibustion in KM therapy. This study aimed to produce practical insights by reviewing patterns of KM use and perceptions over time. Additional surveys must be considered to produce a more in-depth analysis.

**Electronic supplementary material:**

The online version of this article (10.1186/s12906-017-1969-8) contains supplementary material, which is available to authorized users.

## Background

Korea has its own traditional medicine, similar to other countries in Asia, Europe, and Africa. Although Korean medicine (KM) was greatly influenced by traditional Chinese medicine, Koreans have developed their own medical theories, pathologies, methods of diagnosis, and treatment methods [1–5]. Korea has a unique medical system in which both Western medicine (also called “conventional medicine”) and KM are practiced legally. Most KM practitioners frequently use indigenous therapies to treat patients in their clinics.

The growth of public interest in and the use of KM have been well documented in numerous studies. In addition, several studies have identified characteristics of KM users [6–8] and determinants of KM use [9, 10]. According to our previous study, the popularity of KM among the general public increased from 45.8 to 69.3% between 2008 and 2011 [11, 12]. KM use is more common among females, middle-aged individuals (50s), and highly educated people. In another study, however, region of residence and disease type had more influence on KM use [10]. Similarly, KM prevalence estimates were seriously affected by differences in methodology, such as sampling techniques, survey instruments, data generation and collection [13], and the operating agent. Therefore, repeated surveys at regular intervals with consistent and high-quality methods are recommended to produce detailed information regarding KM use.

The aim of this study was to assess the general perceptions of KM, to investigate the patterns of its usage in 2014, and to identify any changes between 2011 and 2014. This study might help elucidate the degree to which KM is integrated into people’s lives and the factors that are important for KM development.

## Methods

### Questionnaire

A questionnaire was initially developed by Shin et al. [12] and was modified by Woo et al. [11]. In brief, the survey instrument included questions regarding KM utilization; self-perception; reasons for choosing KM modalities, such as acupuncture, moxibustion, cupping, physical therapy, herbal medicine, *Chuna* (including chiropractic and any type of manual therapy primarily designed to address or relieve back pain), and others; and the frequency with which the respondent visited KM clinics. The participants’ trust in KM was evaluated by a questionnaire scale with a total of five questions that comprised two items (KM and Western medicine; 1 = very distrusted, 2 = some distrust, 3 = neutral, 4 = some trust, and 5 = very trusted). Some questions were designed to allow multiple answers to ensure the accuracy of the data that were collected. In brief, single-answer questions were designed to elicit simple responses such as “yes” or “no”, or clear answers such as “KM clinic visit number”. In contrast, multiple-answer questions were designed to elicit complex responses or the respondent’s opinions, such as “reasons for trust/distrust of KM” (Additional file 1).

### Participants

We conducted an online-based survey between November and December 2014 in South Korea. This study did not require institutional review board approval because it was an investigative study that did not involve any interventions (Additional file 2). However, we did receive verbal informed consent from all of the participants and maintained their privacy and anonymity. The survey was administered via MACROMILL EMBRAIN (http://www.embrain.com), one of the largest survey companies in South Korea, which engaged 1,171,800 panelists at the time of our survey. We designed the survey with a target sample of 1000 of these panelists by applying proportional quota sampling to those asked to participate in the study. Eligibility was limited to those who were 20 years of age or older. Among the respondents, 498 (49.8%) were male and 502 (50.2%) were female. The participants were distributed across the country in five large metropolitan cities in South Korea: Seoul, Daejeon, Daegu, Pusan, and Gwangju. The web-based questionnaire was provided to the participants with a cover letter explaining the purpose of the survey.

### Comparison of earlier surveys with our 2014 survey

This type of study was initially designed in 2008 by Dr. HK Shin’s team, who covered real KM modalities in their instrument, unlike other similar surveys that contained a variety of non-KM modalities [12]. Shin’s team conducted a cross-sectional study employing both online surveys and face-to-face interviews and found a 45.8% KM usage rate in 2008. Our team followed the same protocol, except for minor modifications, in 2011 [11]. We added questions regarding KM trust and employed a single online survey. We found a 69.3% KM usage rate and a 66.6% KM trust rate. There were some differences between the two studies. While the 2008 survey was mainly focused on the occurrence of KM adverse events, the 2011 survey was mainly focused on changes in the pattern of KM usage over 3 years. In the present study, we used the same survey methods used in the 2011 survey to minimize heterogeneity between the surveys, as we aim to investigate recent trends in KM usage and changes in trust rates.

### Statistical analysis

All of the data collected in the survey were analyzed using SAS 9.3 (SAS Institute Inc., Cary, NC, USA). The data were assessed using Pearson’s chi-square test to compare categorical variables between KM use and the socio-demographic characteristics of the respondents. Student’s t-test was applied to assess continuous variables.

## Results

### Reduction in KM trust, particularly among men and younger people

**Fig. 1:**
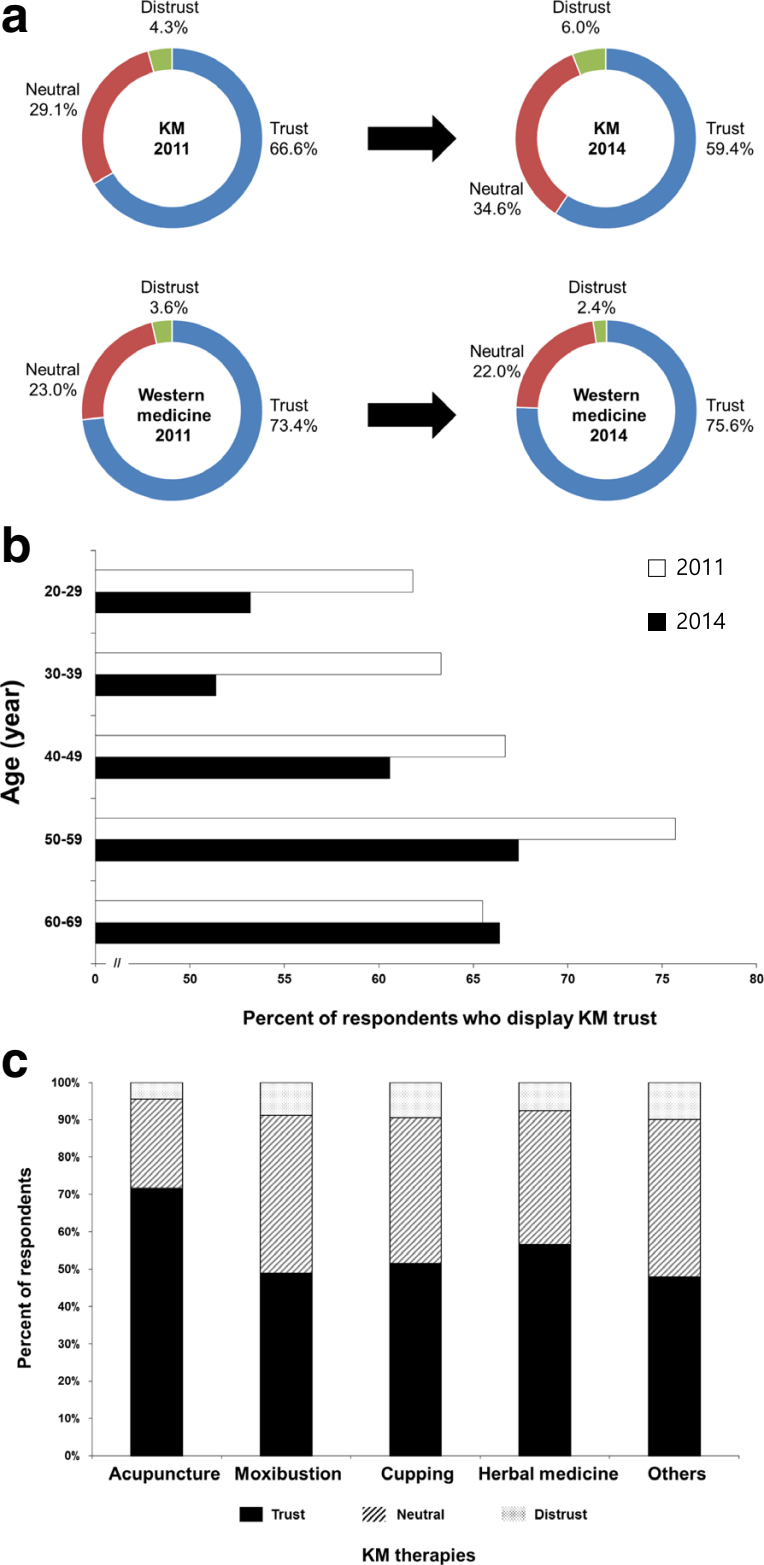
Reduction in KM trust between 2011 and 2014**. a** KM trust decreased from 66.6 to 59.4%. By contrast, trust in Western medicine was nearly the same between 2011 and 2014. **b** The decrease in KM trust appeared in all age groups, except for participants in their 60s. Favorable perceptions were reduced more significantly among members of the general public in their 20s and 30s than in any other age groups. The 2011 survey data were obtained from Woo et al. [11]. **c** The respondents perceived that acupuncture was the most trustable among the KM therapies. Regarding other KM therapies, approximately half of the participants perceived moxibustion, cupping, and KM herbal medicines as trustable

Table 1 shows the socio-demographic characteristics of the respondents in this survey. Compared to the 2011 survey, the survey methods and demographic profiles were largely equivalent. In both surveys, the data were collected from participants living in five large metropolitan cities in South Korea distributed evenly across the country: Seoul, Daejeon, Daegu, Busan, and Gwangju. In 2014, the majority of the respondents trusted both KM and Western medicine. Women (61.8%) trusted KM slightly more than men (57.0%). People aged 50 to 59 reported higher rates of KM trust (67.4%) compared with older and younger people. The respondents who trusted KM tended to trust Western medicine as well (87.0%). In particular, most respondents who distrusted KM trusted Western medicine (65.0%), although a few respondents who distrusted Western medicine also distrusted KM (15.0%) (Table 2). Compared to the 2011 survey, KM trust decreased significantly from 66.6 to 59.4%. The respondents who showed a neutral attitude toward KM appeared to compensate for the gap. There was a remarkable decrease in trust of KM among men (66.5% in the 2011 survey and 57.0% in the 2014 survey). In contrast, trust in Western medicine was largely equal between 2011 and 2014 (Fig. 1a). Regarding participants’ age, similar to the 2011 survey, there was a positive trend in the respondents’ favorable attitudes toward KM as age increased. A decrease in the trust of KM appeared in all age groups, except for participants in their 60s. Favorable perceptions of KM by individuals in the general public in their 20s and 30s were reduced more significantly than in any other age group (Fig. 1b) (Additional file 3). The participants were asked how trustable KM therapies were. As shown in Fig. 1c, acupuncture was considered the most trustable of the KM therapies (71.7%). Approximately half of the participants regarded moxibustion, cupping, and KM herbal medicines to be trustable: 48.9% for moxibustion, 51.5% for cupping, and 56.6% for KM herbal medicines.

**Table 1:** Survey methods and demographic profiles of the 2011 survey and the 2014 survey

	2011 survey *Reference* [11]	2014 survey
Survey methods		
No. of questionnaire items	18 questions	20 questions (2 questions were added)
Data collection method	Online	Online
Sample size	1000	1000
Demographics		
Gender		
Male	499	498
Female	501	502
Age (yr)		
20–29	207	190
30–39	237	222
40–49	231	236
50–59	206	221
60–69	119	131
Region of residence		
Seoul	541	538
Daejeon	79	77
Daegu	125	128
Busan	182	186
Gwangju	73	71

**Table 2:** Trust in KM and Western medicine in 2014

Characteristics	KM trust (%)				Western medicine trust (%)			
	Trust	Neutral	Distrust	*P*-value	Trust	Neutral	Distrust	*P*-value
(*Average*)	(*59.4*)	(*34.6*)	(*6.0*)		(*75.6*)	(*22.0*)	*(2.4*)	
Gender				0.133				0.907
Male	57.0	35.7	7.2		75.7	21.7	2.6	
Female	61.8	33.5	4.8		75.5	22.3	2.2	
Age (yr)				<0.001				0.439
20–29	53.2	38.4	8.4		74.2	23.2	2.6	
30–39	51.4	40.1	8.6		76.6	21.6	1.8	
40–49	60.6	34.3	5.1		74.2	22.9	3.0	
50–59	67.4	28.5	4.1		76.5	21.7	1.8	
60–69	66.4	30.5	3.1		77.1	19.8	3.1	
Region of residence				0.730				0.440
Seoul	59.1	33.8	7.1		76.2	21.4	2.4	
Daejeon	61.0	31.2	7.8		74.0	24.7	1.3	
Daegu	57.8	35.2	7.0		74.2	21.1	4.7	
Busan	62.9	34.9	2.2		74.2	23.7	2.2	
Gwangju	53.5	42.3	4.2		78.9	21.1	0.0	
KM trust								<0.001
Trust					87.0	11.8	1.2	
Neutral					57.8	39.9	2.3	
Distrust					65.0	20.0	15.0	
Western medicine trust				<0.001				
Trust	68.4	26.5	5.2					
Neutral	31.8	62.7	5.5					
Distrust	29.2	33.3	37.5					

Averages are represented as (*n*). The values represent percentages of participants. A comparison with the 2011 data is provided in Table 1 of Additional file 3

### Perceptional differences in reasons for trust/distrust of KM by gender and age

For the participants who expressed that KM was trustworthy (*n* = 594), the survey asked about their reasons for trusting KM. Because it was predicted that several reasons might apply, the question was structured to allow multiple answers. The primary reasons for KM trust were that it has a low rate of side effects (52.7%) and that it has been utilized by people for hundreds of years (48.7%). The other reasons were effectiveness (29.6%), comfort (19.0%), and positive reputation (8.9%). There was no large difference between men and women. There was an age-based difference, however: older people cited “low rate of side effects” far more often than younger people (Table 3). The survey also asked those respondents who expressed that KM was not trustworthy regarding the reasons for their distrust of KM (*n* = 406). There were some differences according to gender and age. “Lack of scientific evidence” was the most common reason in men (59.3%), followed by “unstandardized clinic protocol” (36.4%). However, “suspicion of KM safety” was most common in women (47.4%), followed by “lack of scientific evidence” (44.3%). Additionally, young people aged 20 to 39 years cited “lack of scientific evidence” as the most common reason, whereas older people aged 60 to 69 years cited “suspicion of KM safety” as the most common reason. Other reasons included “bad reputation”, “high prices”, and “high rate of adverse effects” (Table 4). With regard to the 2011 survey, the average percentage of participants citing “lack of scientific evidence” nearly tripled, from 17.1% to 52.2%. Moreover, the percentage of “low efficacy” more than doubled, from 11.7% to 25.9%. In comparison, the percentage of “suspicion of KM safety” decreased slightly, from 46.9% to 35.7% (Additional file 3).

**Table 3:** Reasons for trusting KM therapies in 2014

Category	Low rate of side effects	Proven cures for hundreds of years	Effectiveness	Comfort	Positive reputation	Doctors’ good explanations	Accessibility of KM information to the public	Others	*P*-value
(*Average*)	*(52.7*)	(48.7)	(29.6)	(19.0)	(8.9)	(8.4)	(2.2)	(0.7)	0.019
Gender									
Male	52.8	57.4	29.9	19.0	8.1	7.7	2.1	0.4	
Female	52.6	40.6	29.4	19.0	9.7	9.0	2.3	1.0	
Age (yr)									0.012
20–29	38.6	41.6	36.6	23.8	15.8	2.0	2.0	2.0	
30–39	49.1	42.1	30.7	19.3	10.5	10.5	4.4	0.0	
40–49	51.0	49.7	32.2	16.8	8.4	11.9	2.1	0.0	
50–59	60.4	57.0	24.8	17.4	5.4	8.1	2.0	0.0	
60–69	63.2	49.4	24.1	19.5	5.7	8.0	0.0	2.3	

The 594 respondents who cited KM trust were surveyed. The questions allowed multiple answers. The values represent percentages of participants

**Table 4:** Reasons for distrusting KM therapies in 2014

Category	Lack of scientific evidence	Suspicious of KM safety	Unstandardized clinical protocol	Low efficacy	Unfamiliarity of KM therapies	Irrational KM doctors’ explanations	Discomfort with practice	Others	*P*-value
(*Average*)	*(52.2*)	(*35.7*)	(*35.5*)	(*25.9*)	(*15.8*)	(*7.9*)	(*6.4*)	(*5.2*)	<0.001
Gender									
Male	59.3	25.2	36.4	25.2	19.2	7.9	7.5	5.6	
Female	44.3	47.4	34.4	26.6	12.0	7.8	5.2	4.6	
Age (yr)									0.047
20–29	49.4	19.1	43.8	20.2	20.2	10.1	7.9	7.7	
30–39	57.4	23.1	37.0	31.5	13.0	9.3	6.5	5.6	
40–49	52.7	41.9	32.3	26.9	18.3	5.4	7.5	2.2	
50–59	54.2	55.6	30.6	25.0	6.9	9.7	4.2	1.4	
60–69	40.9	54.5	29.5	22.7	22.7	2.3	4.5	6.8	

The 406 respondents who cited KM distrust were surveyed. The questions allowed for multiple answers. The values represent percentages of participants

### Decline in KM clinic visits in all age groups compared to the 2011 survey

**Fig. 2:**
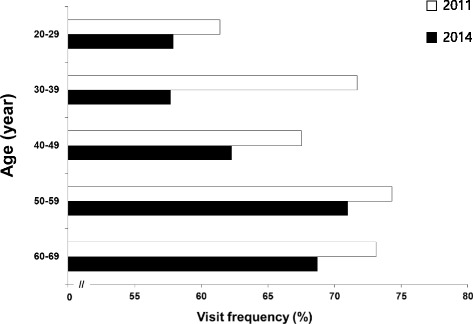
Reduction in TK clinic visits by age between 2011 and 2014. In 2014, there were larger decreases in visit frequency among participants their 20s and 30s than in the other age groups compared to the 2011 survey. The 2011 survey data were obtained from Woo et al. [11]

Table 5 shows the visit frequency to KM clinics over the past 12 months. Of the participants, 63.2% had visited KM clinics at least once during the past 12 months. Less than half (48.9%) of the general population had visited KM clinics between one and four times, whereas 6.4% of the general population had used KM therapies more than 10 times in the past year. Females were slightly more likely to have visited a KM clinic than males, although the total visit frequency had decreased between the two surveys in both men (65.3% visited in the 2011 survey versus 60.2% in the 2014 survey) and women (73.3% visited in the 2011 survey versus 66.1% in the 2014 survey) (Additional file 3). The age distribution of the KM users peaked in the 50s, followed by the 60s and 40s, whereas the age distribution for having had more than 10 visits showed a peak in the 60s, followed by the 50s. KM trust was highly correlated with the frequency of visits to KM clinics by gender and age. The sharpest decline in visit frequency was in participants in their 30s, which correlated with the largest drop in KM trust (Fig. 2). Moreover, even participants in their 60s, who showed an increase in KM trust from the 2011 survey, showed decreased visit frequency (73.1% visited in the 2011 survey versus 68.7% in the 2014 survey).

**Table 5:** Frequency of visits to KM clinics over the past 12 months in 2014

Category	Non-user (%)	User (%)				*P*-value
		Total	No. of KM visits			
			1–4	5–9	≥10	
(*Average*)	(*36.8*)	(*63.2*)	(*48.9*)	(*7.9*)	(*6.4*)	0.024
Gender						
Male	39.8	60.2	48.8	7.0	4.4	
Female	33.9	66.1	49.0	8.8	8.4	
Age (yr)						0.089
20–29	42.1	57.9	47.4	6.3	4.2	
30–39	42.3	57.7	44.1	7.2	6.3	
40–49	37.7	62.3	50.0	8.1	4.2	
50–59	29.0	71.0	52.9	9.5	8.6	
60–69	31.3	68.7	50.4	8.4	9.9	
Region of residence						0.705
Seoul	37.7	62.3	48.3	7.4	6.5	
Daejeon	33.8	66.2	51.9	7.8	6.5	
Daegu	36.7	63.3	53.1	7.0	3.1	
Busan	37.1	62.9	45.7	8.1	9.1	
Gwangju	32.4	67.6	50.7	12.7	4.2	
KM trust						0.000
Trust	28.1	71.9	52.9	10.3	8.8	
Neutral	47.1	52.9	44.8	4.6	3.5	
Distrust	63.3	36.7	33.3	3.3	0.0	
Western medicine trust						0.586
Trust	35.6	64.4	50.3	7.8	6.3	
Neutral	41.8	58.2	43.2	8.2	6.8	
Distrust	29.2	70.8	58.3	8.3	4.2	

Averages are represented as (*n*). The values represent percentages of participants. A comparison of the corresponding 2011 data is shown in Table 2 of Additional file 3

### Decreased usage of moxibustion and cupping and increased usage of herbal medicines

**Fig. 3:**
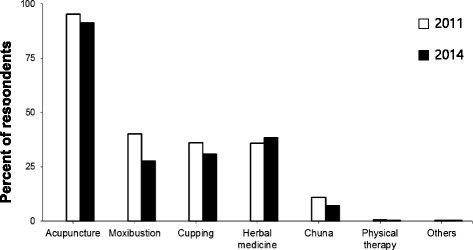
Changes in the usage of KM therapies between 2011 and 2014. The graph shows the changes over time in the patterns of various therapies received in KM clinics. The 2011 survey data were obtained from Woo et al. [11]

Table 6 summarizes the prevalence of different KM therapies in 2014. Among the KM clinic visitors, most received acupuncture, moxibustion, cupping, and herbal medicines. Regardless of gender, age, region of residence, or perceived KM trust, most participants who had experienced KM therapies received acupuncture. Compared with the 2011 survey, the percentages of those who received moxibustion or cupping therapies decreased, whereas the percentage of those who received herbal medicine slightly increased (Additional file 3). In particular, the usage of moxibustion or cupping by older participants in their 40s to 60s was decreased. Figure 3 describes the prevalence of KM therapies in 2011 and 2014. The results showed that the use of herbal medicines had increased slightly, whereas the use of other therapies had decreased over time.

**Table 6:** Prevalence of the use of KM therapies in 2014

Category	Acupuncture	Moxibustion	Cupping	Herbal medicines	Chuna*	Physical therapy	Others	*P*-value
*(Average)*	(*91.2*)	(*27.6*)	(*30.8*)	(*38.2*)	(*7.1*)	(*0.2*)	(*0.2*)	
Gender								0.523
Male	90.9	26.7	28.8	34.7	6.7	0.0	0.0	
Female	91.5	28.5	32.6	41.4	7.5	0.3	0.3	
Age (yr)								0.316
20–29	86.7	28.6	28.6	37.1	3.8	1.0	0.0	
30–39	95.7	29.1	41.0	36.8	8.5	0.0	0.0	
40–49	93.0	26.8	26.1	41.5	8.5	0.0	0.7	
50–59	89.5	28.3	31.6	37.5	4.6	0.0	0.0	
60–69	90.9	25.0	26.1	37.5	11.4	0.0	0.0	
Region of residence								0.029
Seoul	92.2	25.2	30.2	41.1	5.3	0.3	0.0	
Daejeon	85.7	20.4	36.7	46.9	8.2	0.0	0.0	
Daegu	92.0	32.0	26.7	26.7	5.3	0.0	1.3	
Busan	92.2	31.3	25.2	38.3	10.4	0.0	0.0	
Gwangju	86.4	36.4	50.0	27.3	13.6	0.0	0.0	
KM trust								0.000
Trust	91.1	27.6	31.4	41.5	7.0	0.2	0.0	
Neutral	92.8	28.7	30.5	31.7	6.6	0.0	0.0	
Distrust	80.0	20.0	20.0	25.0	15.0	0.0	5.0	

Averages are represented as (*n*). The 632 respondents who had experience with KM therapies were surveyed. These questions allowed for multiple answers. The values represent percentages of participants. *Chuna may involve chiropractic, *Chuna*, and other similar manual therapies primarily designed for back pain. A comparison of the corresponding 2011 data is shown in Table 3 of the Additional file 3

## Discussion

Our study was conducted using a three-year periodic survey with consistent measurements to identify KM trends in the general public in South Korea. In this study, we explored the perceptions and use of KM and examined chronological differences. The reasons why the general public chooses KM therapies have been previously discussed but are not fully understood [10, 14]. Previous studies indicated that KM users were more likely to be female, to trust KM, or to have more education [11, 12]. There might be additional complex psycho-social factors associated with the use of KM. It has been reported that patients frequently use KM because they are not satisfied with conventional treatments.

Trust is an essential prerequisite for the effectiveness of healthcare and leads to the specific expectation that human actions will be beneficial rather than detrimental. However, the association between trust in KM and its general use has seldom been reported. Trust in a healthcare system is not a simple concept; it consists of multi-dimensional features based on values, beliefs, experiences, personality, health status, individual needs, the presence of an illnesses or symptom, and KM practitioners’ demeanor [15–19]. Nevertheless, it is worth noting that trust is a very important factor in the decision to visit a KM clinic or to use KM. We found that the rate of trust was positively linked to the usage of KM modalities. Compared to participants who distrusted KM, those who trusted KM were more likely to receive KM therapies. Interestingly, the respondents who distrusted KM therapies were more likely to use *Chuna* (15%) and other therapies (5%) than those who trusted KM. It is likely that the people who distrusted KM therapies preferred externally applied KM therapies such as moxibustion, cupping and *Chuna* to internally applied KM therapies such as acupuncture and herbal medicine.

The most commonly used KM therapies were acupuncture (91.2%), herbal medicine therapy (38.2%), cupping (30.8%), and moxibustion (27.6%). It has been reported that satisfaction and attitudes toward acupuncture among Western-trained medical doctors and patients were favorable [14]. The use of acupuncture has spread to more than 140 countries and regions. More than 800 diseases are treated with acupuncture in clinics around the world [20]. Acupuncture is practiced for the relief or prevention of pain and for various other health conditions [21]. Unexpectedly, our study found a sudden decrease in the use of moxibustion between 2011 and 2014, which might be correlated with low trust in moxibustion. The mechanisms of moxibustion mainly relate to the thermal effects, radiation effects, and pharmacological actions of moxa and its combustion products [22]. Moxa smoke can be used in air disinfection and as an antiviral and antifungal agent [22]. However, there are concerns regarding the safety of moxa smoke. Some reports have shown that moxa smoke may be harmful to the human body, such as causing allergic reactions [22–24]. Moreover, moxibustion requires extreme care due to the risk of burns. Although the decline in the use of moxibustion is difficult to explain, and it is unclear whether this trend is a transient phenomenon, the reason appears multifactorial, including factors such as growing concern about safety and discomfort in usage.

Women have generally shown more favorable attitudes toward alternative medicine than men in most countries [25]. Similarly, women (61.8%) showed more trust than men (57.0%) in our study. However, the overall reasons for trust did not differ substantially by gender (Table 3). It is interesting that the reasons for KM distrust apparently differed by gender. The main reason for distrust of KM among females was “suspicion of KM safety”, which was cited at a rate nearly twice that of men (47.4% in women versus 25.2% in men) (Table 4). This finding is notable because women were more commonly consumers of herbal medicine than men (41.4% of women versus 34.7% of men) (Table 6). This distrust might be attributed to concerns regarding the safety of herbal medicines. KM herbs are prescribed according to formulas from ancestors found in classic literature. Herbal medicines are used to treat disease, enhance general health and restore health. The use of herbal medicines by the general population or patients has increased. However, there is great concern regarding the risk that these herbal medicines are potentially harmful in unknown ways because there remains an incomplete understanding of how herbal substances react in the body. Unfortunately, some KM doctors have insisted on using secret recipes when prescribing herbal medicine formulas for treating specific illnesses. Based on KM doctors’ private clinical experiences, they can omit any part of the treatment described in classic references or add hidden materials to form their own “secret formula” without rigorous safety testing. This is one of the key reasons for KM distrust because women are more sensitive to negative emotions, meaning that women may react differently than men [26]. To shore up public trust, the safety of herbal medicines for various illnesses must be ensured by applying scientific research approaches (scientification). As a good example of how to increase trust, evidence-based medicine (EBM) has been applied to KM, particularly to explore potential benefits and safety. EBM makes the explicit, judicious, and conscientious use of the best evidence to make decisions regarding preventing diseases, promoting recovery, and improving quality of life [27, 28].

We found that the use of KM therapies, which was previously on the rise, has decreased slightly. Overall, the 1-year prevalence of KM use was 66.6% in 2011 and 59.4% in 2014. Significant declines were found in men and younger participants in their 20s and 30s. There was a remarkable decrease in the trust of KM among men (66.5% in the 2011 survey and 57.0% in the 2014 survey). Comparing our results with those of previous KM surveys, we found a much higher prevalence of use by participants in their 20s and 30s than that reported by Shin et al. [12]. However, we also observed a dramatic reduction in KM trust and usage in those generations compared to our previous survey in 2011 [11]. Although the definitive reason is unclear, several unique demographic features and socio-environmental conditions are suggested. First, younger generations are more likely to believe in the importance of scientification or standardization of medicines compared to their parents’ generation. Younger participants who were born between 1977 and 1994 and were 22–39 years of age as of 2016 are called “generation Y” (Gen Y), and potential reasons for their divergence from older generations are the immense technological development and high educational standards of this period. Members of Gen Y react strongly to real-life examples, and they favor the truth and what is real [29, 30]. In contrast, despite enormous scientific efforts, KM is currently not fully understood in terms of its efficacy and adverse effects. The demanding minds of Gen Y may seek medicines that have been more scientifically proven under rigorous healthcare standards. Second, individuals belonging to Gen Y are less economically advantaged than those of other generations because the majority are financially vulnerable in their careers. Currently, a monthly dose of herbal medicine typically costs approximately 400,000 won (US $352). Since late 2011, crude herb prices have increased sharply, up to three-fold, because of decreases in imports from China. Furthermore, KM therapies are not fully covered under national health insurance. High prices may result in an out-of-pocket economic burden, particularly for those who are most vulnerable to financial strains, although other patients are less price sensitive if they believe they are receiving superior medical services and good products. Third, Gen Y individuals have different motivations for medical consumption and purchase engagement. They are reported to be loyal online customers and to show greater confidence and trust in the brand names of their choice compared to all other generations [31, 32]. These younger consumers may be attracted to easier and more convenient purchase options. In fact, they are well accustomed to internet shopping, even for healthcare products. The online/wireless markets related to health foods grew steadily between 2011 and 2014. During this time, Gen Y was the main force behind online shopping [33, 34]. However, the expansion of internet health food markets is one of the greatest challenges facing KM because these markets are becoming alternatives to KM modalities [35].

There were several limitations to our survey. First, we included only six major KM modalities in this survey to follow the same measurements used in the 2011 survey. Because the main focus of our study was the comparison between the two surveys, as previously stated, we did not provide many options for respondents to mention other KM modalities. Second, the data were based entirely on laypeople’s self-reporting within a specified period. Therefore, there is potential recall bias. Furthermore, our approach was cross-sectional, indicating that the sample used different panels from the 2011 survey. Because our survey instruments were mostly determined by the 2011 survey, we did not evaluate validity or reliability. However, we consulted with several experts to determine whether the questions were clear, understandable, and presented in a logical order prior to conducting the survey.

## Conclusion

Although the majority of the Korean population trusted KM, the frequency of visits to KM clinics decreased from 66.6 to 59.4% between 2011 and 2014. A reduction in KM use was particularly noted in younger adults. Distrust was most likely related to the lack of scientific evidence or suspicion of KM safety, implying that future improvements in the understanding and knowledge of KM may lead to positive outcomes, such as higher trust, expanded prevalence, and more sustainable development. By acquiring KM patterns over time, this periodic study produced beneficial practical insights. For an in-depth analysis, additional surveys must be considered in the future.

## Additional files

Additional file 1: Questionnaire to investigate the attitude toward Korean Medicine among the public in South Korea (DOCX 21 kb)

Additional file 2: Standard Operation Principles for Clinical Trials and Institutional Review Board (IRB) operation (DOCX 189 kb)

Additional file 3: **Table S1.** Demographics of respondents and trust of KM and Western medicine in 2011 and 2014 (DOCX 37 kb)

## Abbreviations

CPG: Clinical practice guidelines
EBM: Evidence-based medicine
Gen Y: Generation Y
KM: Korean medicine
R&D: Research and development
