# Supplementary material for: Changes in trust and the use of Korean medicine in South Korea: a comparison of surveys in 2011 and 2014
Source: BMC Complement Altern Med. 2017 Sep 16;17:463. doi: 10.1186/s12906-017-1969-8 (PMC5603087; doi:10.1186/s12906-017-1969-8)
Supplement: Supplementary file 1 — Questionnaire to investigate the attitude toward Korean Medicine among the public in South Korea (DOCX 21 kb) [file 12906_2017_1969_MOESM1_ESM.docx]

*Please note that this questionnaire is translated from the original Korean version.*

**Questionnaire to investigate the attitude toward Korean Medicine among the public in South Korea**

We thank you for taking the time to fill out this questionnaire. Your answers and comments will provide us with a greater understanding of the Korean Medicine (KM) trend and help to develop a more effective scientific planning. This questionnaire is approved by the ethical review committee of the Korea Institute of Oriental Medicine, South Korea.

**Definitions:**

***Korean Medicines*** refers to traditional medical field developed and practiced in Korea.

***Acupuncture*** is the stimulation of specific points along the skin of the body involving various methods such as the application of heat, pressure, or laser or penetration of thin needles.

***Cupping*** involves warming the air inside a glass, metal, or wooden cup and inverting it over a part of the body to treat various health conditions.

Moxibustion involves the burning of mugwort, a small, spongy herb, to facilitate healing.

***Chiropractic*** is a health care profession that focuses on disorders of the musculoskeletal system and the nervous system, and the effects of these disorders on general health.

***Physical therapy*** is a health care profession primarily concerned with the remediation of impairments and disabilities and the promotion of mobility, functional ability, quality of life and movement potential through examination, evaluation, diagnosis and physical intervention.

***Traditional herbal medicines*** include herbs, herbal materials, herbal preparations and finished herbal products that contain as active ingredients parts of plants, or other plant materials, or combinations.

Please read and answer the following questions.

**Demographic and other Characteristics**

1. Gender: □ Male □ Female

2. Age (in years): □ 20-29 □ 30-39 □ 40-49 □ 50-59 □ 60-69

3. Marital status: □ Single □ Married □ Divorced

4. Educational level: □ High school graduated □ College □ College graduated

5. Residence: □ Seoul □ Daejeon □ Daegu □ Busan □ Gwangju

6. Monthly Income: □ Less than 200 KW □ 200-500 KW □ Greater than 500 KW

7. Employment: □ Full time □ Part time □ Homemaker □ Retired □ Unemployed

8. Are you still currently working? □ Yes □ No

**KM reliability:**

9. How much do you trust KM?

□ A lot of (5 point) □ Some (4 point) □ Neutral (3 point) □ Little (2 point) □ No (1 point)

**If little or no, go to Question 11**

10. Why do you trust KM therapies?

□ Proven cures for hundreds of years

□ Low rate of side effects

□ High reputation

□ Comfort

□ Effectiveness

□ Doctor’s good explanation

□ Opened KM information to the public

□ Other (please specify) …………………

11. Why do you distrust KM therapies?

□ Lack of scientific evidence

□ Suspicious of KM safety

□ Unfamiliarity of KM therapies

□ Discomfort practice

□ Unstandardized clinic protocol

□ Low efficacy

□ Low reputation

□ Other (please specify) …………………

12. How much do you trust Western medicine?

□ A lot of (5 point) □ Some (4 point) □ Neutral (3 point) □ Little (2 point) □ No (1 point)

**Reason for visit:**

13. How often during the last year have you visited KM clinics?

□ Never

□ 1 to 4 times

□ 5 to 9 times

□ 10 or more

**If never, you do not need to continue. Thank you for answering this questionnaire.**

14. Which KM therapy have you used? Please select all that apply

□ Acupuncture

□ Cupping

□ Moxibustion

□ Chiropractic

□ Physical therapy

□ Traditional herbal medicines

□ Other (please specify) …………………

15. Why did you decide to use KM therapies? Please select all that apply

□ To get health consultation

□ To take physical therapy

□ To take traditional herbal medicine

□ To receive therapeutic treatments

□ To vaccinate

□ Other (please specify)…………………

16. Who recommended you the use of KM therapies you had taken at first?

□ Medical doctor

□ Parents or friends

□ Media (TV/Newspaper)

□ Pharmacist

□ Other (please specify)…………………

**Others:**

17. Have you ever experienced any adverse effect from KM therapies?

□ Yes □ No □ Don’t know

18. Can you estimate the average cost per year of KM?

□ ( kw)

19. Which part of KM should be focused on promoting development?

□ Evidence-based effectiveness

□ Evidence-based safeness

□ Development of new KM products

□ Generalization of KM therapeutic approaches

□ Theories of KM

□ Other (please specify) …………………

20. What is most necessary for the sustainable KM promotion in the future?

|  |
| --- |

**Thank you for your participation in this survey.**
