# Supplementary material for: Changes in trust and the use of Korean medicine in South Korea: a comparison of surveys in 2011 and 2014
Source: BMC Complement Altern Med. 2017 Sep 16;17:463. doi: 10.1186/s12906-017-1969-8 (PMC5603087; doi:10.1186/s12906-017-1969-8)
Supplement: Supplementary file 2 — Standard Operation Principles for Clinical Trials and Institutional Review Board (IRB) operation (DOCX 189 kb) [file 12906_2017_1969_MOESM2_ESM.docx]

*Please note that this regulation is translated from the original Korean version.*

**Standard Operation Principles for Clinical Trials and Institutional Review Board (IRB) operation**

(Ⅲ-3-5)

**Chapter 4. Responsibilities and authorities of Institutional Review Board (hereinafter referred to as "IRB")**

**Article 27 (Review exemption subjects)**

**(1)** Review exemption is allowed for followings, when a human study has little harmful effect on study subjects and public;

1. A study that directly intervenes subjects or subjects’ environments, and satisfies one of followings;

1-2. A study without invasive procedures such as drug administration or blood sampling

1-3. A study that only utilizes instruments for simple measurement or observation without systemic effect

1-4. A quality or taste evaluation study with food that is allowed to be commercialized under article 3 of the Law of Food Sanitation.

**2. Study that evaluates general population (whether it is face-to-face interview or not) and does not collect or record private information, under article 23 of the Privacy Protection Law**.

3. Study that utilizes previous subject data or documents

4. Study that utilizes public information

**(2)** Review exemption of human derivatives study is allowed for followings;

1. Study that received human derivatives from the bank. An investigator does not collect or record any identification of subjects. An access to subjects’ identification is only available through human derivatives bank.

2. Study that does not directly collect human derivatives, but utilizes investigational materials (pathogen, cell line etc.) those were collected or managed from human derivatives so that also public population utilize it (excluding embryonic stem cell).

3. Study that was executed or approved by country, when there is any urgent threatening of public health. The Health Authority (HA) minister allows review exemption. Afterward, the report should be submitted to public authority that was designated by HA minister.

**(3)**  Despite of paragraph (1), a study that utilizes subjects under vulnerable environment (the Pharmaceutical Affairs Law, No 3-2) is not eligible for review exemption.

**(4)** Despite of paragraph (1), an investigator could request IRB review when it supposed to be necessary for study operation.

*The original Korean version*

**
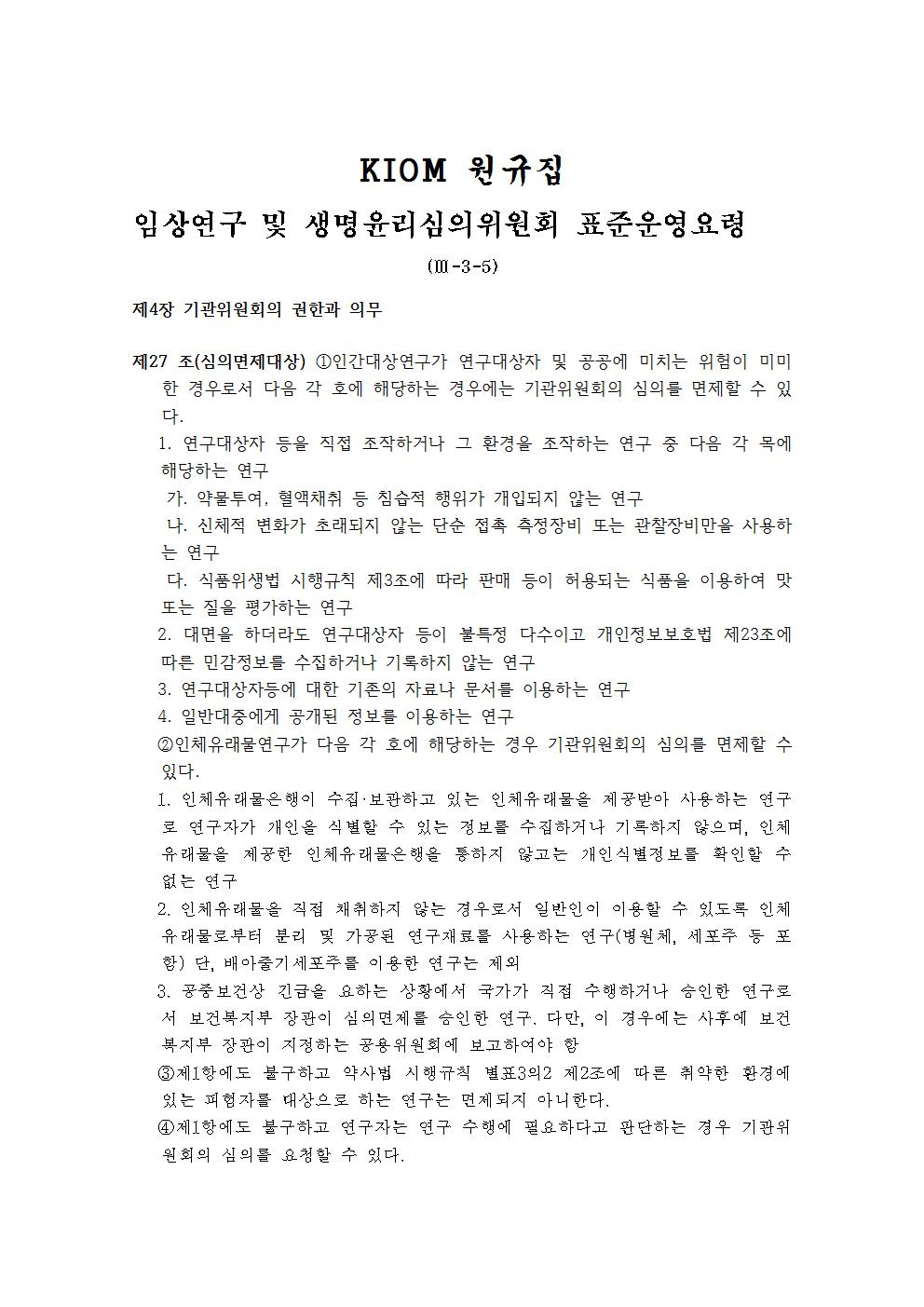
**
