# Supplementary material for: Changes in trust and the use of Korean medicine in South Korea: a comparison of surveys in 2011 and 2014
Source: BMC Complement Altern Med. 2017 Sep 16;17:463. doi: 10.1186/s12906-017-1969-8 (PMC5603087; doi:10.1186/s12906-017-1969-8)
Supplement: Supplementary file 3 — Table S1. Demographics of respondents and trust of KM and Western medicine in 2011 and 2014 (DOCX 37 kb) [file 12906_2017_1969_MOESM3_ESM.docx]

**Additional file 3: Table S1. Demographics of respondents and trust of KM and Western medicine in 2011 and 2014**

|  | 2011  *Reference* [[11](#_ENREF_11)] | | | | | | | |  | 2014 | | | | | | | | |
| --- | --- | --- | --- | --- | --- | --- | --- | --- | --- | --- | --- | --- | --- | --- | --- | --- | --- | --- |
|  | KM trust (%) | | | | Western medicine trust (%) | | | |  | KM trust (%) | | | | | Western medicine trust (%) | | | |
| Characteristics | Trust | Neutral | Distrust | *P*-value | Trust | Neutral | Distrust | *P*-value |  | Trust | Neutral | Distrust | *P*-value | Trust | | Neutral | Distrust | *P*-value |
| (*Average*) | (66.6) | (29.1) | (4.3) |  | (73.4) | (23.0) | (3.6) |  |  | (*59.4*) | (*34.6*) | (*6.0*) |  | (*75.6*) | | (*22.0*) | *(2.4*) |  |
| Gender |  |  |  | 0.515 |  |  |  | 0.373 |  |  |  |  | 0.133 |  | |  |  | 0.907 |
| Male | 66.5 | 28.5 | 5.0 |  | 75.2 | 21.8 | 3.0 |  |  | 57.0 | 35.7 | 7.2 |  | 75.7 | | 21.7 | 2.6 |  |
| Female | 66.7 | 29.7 | 3.6 |  | 71.7 | 24.2 | 4.2 |  |  | 61.8 | 33.5 | 4.8 |  | 75.5 | | 22.3 | 2.2 |  |
| Age (yr) |  |  |  | 0.029 |  |  |  | 0.419 |  |  |  |  | <0.001 |  | |  |  | 0.439 |
| 20-29 | 61.8 | 30.9 | 7.2 |  | 69.6 | 27.1 | 3.4 |  |  | 53.2 | 38.4 | 8.4 |  | 74.2 | | 23.2 | 2.6 |  |
| 30-39 | 63.3 | 33.8 | 3.0 |  | 72.2 | 24.5 | 3.4 |  |  | 51.4 | 40.1 | 8.6 |  | 76.6 | | 21.6 | 1.8 |  |
| 40-49 | 66.7 | 28.6 | 4.8 |  | 74.0 | 20.3 | 5.6 |  |  | 60.6 | 34.3 | 5.1 |  | 74.2 | | 22.9 | 3.0 |  |
| 50-59 | 75.7 | 22.3 | 1.9 |  | 77.7 | 19.9 | 2.4 |  |  | 67.4 | 28.5 | 4.1 |  | 76.5 | | 21.7 | 1.8 |  |
| 60-69 | 65.5 | 29.4 | 5.0 |  | 73.9 | 23.5 | 2.5 |  |  | 66.4 | 30.5 | 3.1 |  | 77.1 | | 19.8 | 3.1 |  |
| Region of residence |  |  |  | 0.475 |  |  |  | 0.006 |  |  |  |  | 0.730 |  | |  |  | 0.440 |
| Seoul | 68.8 | 26.6 | 4.6 |  | 75.8 | 22.0 | 2.2 |  |  | 59.1 | 33.8 | 7.1 |  | 76.2 | | 21.4 | 2.4 |  |
| Daejeon | 62.0 | 31.6 | 6.3 |  | 73.4 | 16.5 | 10.1 |  |  | 61.0 | 31.2 | 7.8 |  | 74.0 | | 24.7 | 1.3 |  |
| Daegu | 67.2 | 31.2 | 1.6 |  | 66.4 | 30.4 | 3.2 |  |  | 57.8 | 35.2 | 7.0 |  | 74.2 | | 21.1 | 4.7 |  |
| Busan | 64.8 | 31.3 | 3.8 |  | 69.2 | 25.8 | 4.9 |  |  | 62.9 | 34.9 | 2.2 |  | 74.2 | | 23.7 | 2.2 |  |
| Gwangju | 58.9 | 35.6 | 5.5 |  | 78.1 | 17.8 | 4.1 |  |  | 53.5 | 42.3 | 4.2 |  | 78.9 | | 21.1 | 0.0 |  |
| KM trust |  |  |  | <0.001 |  |  |  | <0.001 |  |  |  |  |  |  | |  |  | <0.001 |
| Trust |  |  |  |  | 85.6 | 13.7 | 0.8 |  |  |  |  |  |  | 87.0 | | 11.8 | 1.2 |  |
| Neutral |  |  |  |  | 52.2 | 40.5 | 7.2 |  |  |  |  |  |  | 57.8 | | 39.9 | 2.3 |  |
| Distrust |  |  |  |  | 27.9 | 48.8 | 23.3 |  |  |  |  |  |  | 65.0 | | 20.0 | 15.0 |  |
| Western medicine trust |  |  |  | <0.001 |  |  |  | <0.001 |  |  |  |  | <0.001 |  | |  |  |  |
| Trust | 77.7 | 20.7 | 1.6 |  |  |  |  |  |  | 68.4 | 26.5 | 5.2 |  |  | |  |  |  |
| Neutral | 39.6 | 51.3 | 9.1 |  |  |  |  |  |  | 31.8 | 62.7 | 5.5 |  |  | |  |  |  |
| Distrust | 13.9 | 58.3 | 27.8 |  |  |  |  |  |  | 29.2 | 33.3 | 37.5 |  |  | |  |  |  |

**Table 2. Visit frequency to KM clinics over the past 12 months in 2011 and in 2014**

| year | 2011  *Reference* [11] | | | | | |  | 2014 | | | | | |
| --- | --- | --- | --- | --- | --- | --- | --- | --- | --- | --- | --- | --- | --- |
| Category | Non-user  (%) | User (%) | | | | *P*-value |  | Non-user  (%) | User (%) | | | | *P*-value |
|  |  | Total | No. of KM visits | | |  |  |  | Total | No. of KM visits | | |  |
|  |  |  | 1-4 | 5-9 | ≥10 |  |  |  |  | 1-4 | 5-9 | ≥10 |  |
| (*Average*) | (30.7) | (69.3) | (53.4) | (9.3) | (6.6) | 0.004 |  | (*36.8*) | (*63.2*) | (*48.9*) | (*7.9*) | (*6.4*) | 0.024 |
| Gender |  |  |  |  |  |  |  |  |  |  |  |  |  |
| Male | 34.7 | 65.3 | 51.5 | 9.4 | 4.4 |  |  | 39.8 | 60.2 | 48.8 | 7.0 | 4.4 |  |
| Female | 26.7 | 73.3 | 55.3 | 9.2 | 8.8 |  |  | 33.9 | 66.1 | 49.0 | 8.8 | 8.4 |  |
| Age (yr) |  |  |  |  |  | 0.054 |  |  |  |  |  |  | 0.089 |
| 20-29 | 38.6 | 61.4 | 49.8 | 5.8 | 5.8 |  |  | 42.1 | 57.9 | 47.4 | 6.3 | 4.2 |  |
| 30-39 | 28.3 | 71.7 | 55.7 | 11.4 | 4.6 |  |  | 42.3 | 57.7 | 44.1 | 7.2 | 6.3 |  |
| 40-49 | 32.5 | 67.5 | 52.4 | 10.0 | 5.2 |  |  | 37.7 | 62.3 | 50.0 | 8.1 | 4.2 |  |
| 50-59 | 25.7 | 74.3 | 57.3 | 8.3 | 8.7 |  |  | 29.0 | 71.0 | 52.9 | 9.5 | 8.6 |  |
| 60-69 | 26.9 | 73.1 | 50.4 | 11.8 | 10.9 |  |  | 31.3 | 68.7 | 50.4 | 8.4 | 9.9 |  |
| Region of residence |  |  |  |  |  | 0.305 |  |  |  |  |  |  | 0.705 |
| Seoul | 31.2 | 68.8 | 51.9 | 9.2 | 7.6 |  |  | 37.7 | 62.3 | 48.3 | 7.4 | 6.5 |  |
| Daejeon | 31.6 | 65.4 | 53.2 | 8.9 | 6.3 |  |  | 33.8 | 66.2 | 51.9 | 7.8 | 6.5 |  |
| Daegu | 28.8 | 71.2 | 48.8 | 16.0 | 6.4 |  |  | 36.7 | 63.3 | 53.1 | 7.0 | 3.1 |  |
| Busan | 30.8 | 69.2 | 57.7 | 7.1 | 4.4 |  |  | 37.1 | 62.9 | 45.7 | 8.1 | 9.1 |  |
| Gwangju | 28.8 | 71.2 | 61.6 | 4.1 | 5.5 |  |  | 32.4 | 67.6 | 50.7 | 12.7 | 4.2 |  |
| KM trust |  |  |  |  |  | <0.001 |  |  |  |  |  |  | 0.000 |
| Trust | 25.2 | 74.8 | 54.8 | 11.6 | 8.4 |  |  | 28.1 | 71.9 | 52.9 | 10.3 | 8.8 |  |
| Neutral | 39.9 | 60.1 | 52.6 | 4.8 | 2.7 |  |  | 47.1 | 52.9 | 44.8 | 4.6 | 3.5 |  |
| Distrust | 53.5 | 46.5 | 37.2 | 4.7 | 4.7 |  |  | 63.3 | 36.7 | 33.3 | 3.3 | 0.0 |  |
| Western medicine trust |  |  |  |  |  | 0.038 |  |  |  |  |  |  | 0.586 |
| Trust | 28.1 | 71.9 | 54.8 | 9.9 | 7.2 |  |  | 35.6 | 64.4 | 50.3 | 7.8 | 6.3 |  |
| Neutral | 37.0 | 63.0 | 51.7 | 7.0 | 4.3 |  |  | 41.8 | 58.2 | 43.2 | 8.2 | 6.8 |  |
| Distrust | 44.4 | 55.6 | 36.1 | 11.1 | 8.3 |  |  | 29.2 | 70.8 | 58.3 | 8.3 | 4.2 |  |

**Table 3. Comparison of 2011 and 2014 prevalence of the use of TKM therapies**

| Category | Acupuncture  (%) | |  | Moxibustion  (%) | |  | Cupping  (%) | |  | Traditional herbal medicine  (%) | |  | *Chuna**  (%) | |  | Physical therapy  (%) | |  | Others^†^  (%) | |
| --- | --- | --- | --- | --- | --- | --- | --- | --- | --- | --- | --- | --- | --- | --- | --- | --- | --- | --- | --- | --- |
| Year | 2011 | 2014 |  | 2011 | 2014 |  | 2011 | 2014 |  | 2011 | 2014 |  | 2011 | 2014 |  | 2011 | 2014 |  | 2011 | 2014 |
| (Average) | (95.3) | (*91.2*) |  | (40.1) | (*27.6*) |  | (36.0) | (*30.8*) |  | (35.7) | (*38.2*) |  | (10.9) | (*7.1*) |  | (0.4) | (*0.2*) |  | (0.2) | (*0.2*) |
| Gender |  |  |  |  |  |  |  |  |  |  |  |  |  |  |  |  |  |  |  |  |
| Male | 96.0 | 90.9 |  | 39.6 | 26.7 |  | 35.1 | 28.8 |  | 33.3 | 34.7 |  | 12.0 | 6.7 |  | 0.4 | 0.0 |  | 0.4 | 0.0 |
| Female | 94.7 | 91.5 |  | 40.6 | 28.5 |  | 36.9 | 32.6 |  | 38.1 | 41.4 |  | 9.8 | 7.5 |  | 0.4 | 0.3 |  | 0.0 | 0.3 |
| Age (yrs) |  |  |  |  |  |  |  |  |  |  |  |  |  |  |  |  |  |  |  |  |
| 20-29 | 91.8 | 86.7 |  | 30.6 | 28.6 |  | 28.2 | 28.6 |  | 24.7 | 37.1 |  | 14.1 | 3.8 |  | 0.0 | 1.0 |  | 0.0 | 0.0 |
| 30-39 | 96.4 | 95.7 |  | 34.2 | 29.1 |  | 36.9 | 41.0 |  | 36.9 | 36.8 |  | 11.7 | 8.5 |  | 0.0 | 0.0 |  | 0.0 | 0.0 |
| 40-49 | 95.9 | 93.0 |  | 49.0 | 26.8 |  | 39.8 | 26.1 |  | 34.7 | 41.5 |  | 9.2 | 8.5 |  | 0.0 | 0.0 |  | 0.0 | 0.7 |
| 50-59 | 97.1 | 89.5 |  | 40.8 | 28.3 |  | 39.8 | 31.6 |  | 39.8 | 37.5 |  | 8.7 | 4.6 |  | 1.9 | 0.0 |  | 0.0 | 0.0 |
| 60-69 | 94.4 | 90.9 |  | 47.2 | 25.0 |  | 33.3 | 26.1 |  | 43.1 | 37.5 |  | 11.1 | 11.4 |  | 0.0 | 0.0 |  | 1.4 | 0.0 |
| Region of residence |  |  |  |  |  |  |  |  |  |  |  |  |  |  |  |  |  |  |  |  |
| Seoul | 95.7 | 92.2 |  | 34.9 | 25.2 |  | 33.7 | 30.2 |  | 39.6 | 41.1 |  | 9.8 | 5.3 |  | 0.0 | 0.3 |  | 0.0 | 0.0 |
| Daejeon | 97.0 | 85.7 |  | 48.5 | 20.4 |  | 30.3 | 36.7 |  | 27.3 | 46.9 |  | 18.2 | 8.2 |  | 3.0 | 0.0 |  | 3.0 | 0.0 |
| Daegu | 97.1 | 92.0 |  | 55.9 | 32.0 |  | 45.6 | 26.7 |  | 36.8 | 26.7 |  | 11.8 | 5.3 |  | 1.5 | 0.0 |  | 0.0 | 1.3 |
| Busan | 92.9 | 92.2 |  | 41.2 | 31.3 |  | 37.6 | 25.2 |  | 31.8 | 38.3 |  | 9.4 | 10.4 |  | 0.0 | 0.0 |  | 0.0 | 0.0 |
| Gwangju | 92.9 | 86.4 |  | 35.7 | 36.4 |  | 35.7 | 50.0 |  | 21.4 | 27.3 |  | 14.3 | 13.6 |  | 0.0 | 0.0 |  | 0.0 | 0.0 |
| KM trust |  |  |  |  |  |  |  |  |  |  |  |  |  |  |  |  |  |  |  |  |
| Trust | 96.4 | 91.1 |  | 41.8 | 27.6 |  | 39.1 | 31.4 |  | 38.0 | 41.5 |  | 10.2 | 7.0 |  | 0.6 | 0.2 |  | 0.3 | 0.0 |
| Neutral | 90.9 | 92.8 |  | 36.4 | 28.7 |  | 28.3 | 30.5 |  | 29.3 | 31.7 |  | 14.1 | 6.6 |  | 0.0 | 0.0 |  | 0.0 | 0.0 |
| Distrust | 100.0 | 80.0 |  | 11.1 | 20.0 |  | 0.0 | 20.0 |  | 22.2 | 25.0 |  | 0.0 | 15.0 |  | 0.0 | 0.0 |  | 0.0 | 5.0 |

*Note*. A total of 693 and 632 respondents who had experience with KM therapies were surveyed in 2011 and 2014, respectively. These questions allowed for multiple answers. The values are the percentages of participants. **Chuna* may involve chiropractic, *Chuna*, and other similar manual therapies primarily designed for back pain. The 2011 survey date was referred from Woo et al. [11].
